# Effects of Social Relationships on Mortality among the Elderly in a Japanese Rural Area: An 88-month Follow-up Study

**DOI:** 10.2188/jea.15.78

**Published:** 2005-06-01

**Authors:** Chiyoe Murata, Takaaki Kondo, Yoko Hori, Daiki Miyao, Koji Tamakoshi, Hiroshi Yatsuya, Hisataka Sakakibara, Hideaki Toyoshima

**Affiliations:** 1 Department of Public Health/Health Information Dynamics, Nagoya University Graduate School of Medicine.; 2 Department of Public Health, Hamamatsu University School of Medicine.; 3 Department of Medical Technology, Nagoya University School of Health Sciences.; 4 Department of Nursing, Nagoya University School of Health Sciences.; 5 Department of Public Health and Home Nursing, Nagoya University School of Health Sciences.

**Keywords:** social relationships, living arrangement, multi-generation family, old-old

## Abstract

BACKGROUND: The association between social relationships and lower mortality has been well documented in Western countries. This study aims to investigate that association among elderly Japanese in a rural area.

METHODS: An analysis was conducted with 1,994 subjects (58.1% women), 78.3% of the total elderly aged 65 and older in a town, who were independent in activities of daily living. A baseline survey was carried out in 1992, and subjects were followed until 1999. Cox proportional hazard models examined the association between social relationships (availability of casual friend/ support provider, group membership, job, living arrangement) and an 88-month mortality.

RESULTS: A significant association between social relationships and mortality was observed among the old-old (aged 75 and older). Among men, having a job and group membership were significantly associated with lower mortality with hazard ratios (95% confidence intervals) of 0.62 (0.41-0.94) and 0.60 (0.40-0.90), respectively, after adjustment for age, diagnosed illnesses, self-rated health, other social relationships, annual income, and home ownership. Among women, having a job and living alone were significantly associated with lower mortality with hazard ratios (95% confidence intervals) of 0.67 (0.45-0.99) and 0.35 (0.13-0.97), respectively.

CONCLUSIONS: Social relationships such as having a job and group membership were associated with lower mortality among the old-old. In addition, old-old women living alone were better off in terms of mortality after adjustment for possible confounders. This suggests the importance of considering family relationships in terms of quality in areas where multi-generation households prevail.

The association between social relationships and mortality among the elderly has been well documented by various longitudinal studies.^1^^-^^5^ These studies have found that those with more social ties had a lower risk of mortality, independent of age and other physical or psychological factors.

Examining the association between social relationships and mortality has been a subject of frequent studies in Western nations. However, such studies are relatively few in Asian nations. The purpose of our study is to re-examine the association between social relationships and mortality in a non-Western population, i.e., the elderly in a Japanese rural area. Although the methods of measuring social relationships vary among researchers, two dimensions of social relationships have generally been conceptualized in their approaches.^1^^-^^5^ One approach intended to measure social relationships in terms of social ties or network, i.e., a web of human relations such as friendship or community participation where social interactions occur. The other dealt with the nature of social support such as negative or positive support provided through such interactions. Our study used the first approach, and investigated how social relationships might affect the mortality of community-dwelling elderly in a rural community in Japan.

## METHODS

### Study population

Subjects were all the residents aged 65 and older in Matsukawa, a town in the southern part of Nagano Prefecture in central Japan. With a population of about 14,000, the town residents are mostly engaged in agriculture, tending apple or pear orchards. In our population, 72.5% of men and 48% of women who worked were engaged in agriculture. The average family size was 4.5, which was higher than the Japanese national average of 3.05 in the year 1990^6^ as well as above that of Western nations.^7^ The average life expectancy as of the year 2000 was 79.3 years for men and 85.6 years for women, which was also higher than Japan’s average of 78.0 and 84.9 years in that same year.^8^

In a baseline survey conducted in August of 1992, 88 welfare commissioners distributed self-administered questionnaires consisting of 51 questions regarding self-rated health, diagnosed illnesses or conditions, activities of daily living (ADL), and social relationships. A total of 2,490 of the elderly in the town returned the questionnaire for an overall response rate of 97.7%. The reasons for non-response were hospital stay (n = 42), nursing home stay (n = 8), and others (n = 8). The study objectives were explained to all participants, and confidentiality was assured in written form at the time of the survey. The survey data were collected and made available by the municipality, and the list of participant names and IDs was kept confidential.

### Measurement

Respondents were followed up to the termination of observations due to either death or the end of 1999. The date of death was ascertained by reviewing the local resident registry records. The following five variables were identified as proxies for social relationships: (1) casual friend, (2) support provider, (3) group membership, (4) job, and (5) living arrangements. All of these variables are reported to have significant associations with mortality in various studies.^1^^-^^5^^,^^9^

Availability of casual friends and support providers was derived from the following two questions: “Do you have a friend with whom you can talk frankly?” and “Do you have someone whom you can count on when you are in trouble?”, respectively. The question regarding group membership was: “Are you at present a member of any of the following groups: seniors’ club, neighborhood association, hobby or sports club, religious organization, day service users, association for those living alone, health-oriented learning group, volunteer organization, or any other such group?” Regarding a job, we asked, “Are you engaged in any job?” Job status was defined as having a job regardless of payment or working hours. In a preliminary analysis, when housework was excluded, a job was not associated with women’s mortality in any models. However, in our data, 34.7% of women reported housework as their job, whereas only 1.5% of men did so. Thus, we conducted analyses after redefining a job to include housework. All responses were dichotomized into “yes” or “no,” and respective codes “1” or “0” were assigned. Living arrangement was categorized as the following: “living alone,” “couple only,” “multi-generation,” and treated as a dummy variable with “multi-generation” as the reference category.

As possible confounders, we used age, baseline health status, and socio-economic factors. Regarding health status, we asked if they had any diagnosed illnesses or conditions such as hypertension, heart disease, respiratory disease, gastrointestinal disease, diabetes, stroke, arthritis or neuralgia, eye disease, liver disease, dementia, gynecological disease, or other. For the presence of each illness or condition, the code “1” was assigned, and the un-weighted sum of the count was used in the analysis. Using a simple count of illnesses as an indicator of comorbidity among the elderly has been warranted in various studies.^7^^,^^10^^,^^11^ Self-rated health, which is a strong predictor of mortality among the elderly,^10^^,^^12^ was measured on a 4-point scale of “good,” “average,” “fair,” “poor,” and entered in the model as a continuous variable.

Some studies have reported that socio-economic factors are also related to mortality as well as to social relationships.^13^^-^^15^ We considered these factors as well by using annual income and home ownership as possible confounders. Annual income was categorized into the followings: less than 1 million yen, 1-2 million yen, 2-3 million yen, and 3+ million yen. Home ownership was dichotomized into yes or no.

### Exclusion criteria

Prior to the analyses, we excluded 20 subjects whose mortality status could not be ascertained due to either miscoding or mistranscription or moving out of the subject from the study area. Because they were only 0.8% of the whole respondents and no systematic differences were observed between them and others, we concluded that there was no bias in this regard. Other 344 were eliminated due to missing data on ADL, self-rated health, diagnosed illness under treatment, and social relationships.

In this study, we limited our analyses to the functionally independent elderly in order to eliminate the confounding effect of ADL impairment. In a preliminary analysis, poor ADL proved to be strongly related to social relationships and mortality with Spearman’s correlation coefficient ranging from 0.110 to 0.354 for social relationships and 0.426 for mortality (p<0.001). Hierarchical log-linear analysis also indicated significant (p<0.001) two-way interactions among these variables (see statistical analysis section below for details). Thus, we excluded 132 individuals who needed assistance in any of the following three ADL: bathing, transferring, or using the toilet. Consequently, the analyses were carried out with 1,994 (836 men and 1,158 women) functionally independent subjects, accounting for 80.1% of the total respondents and 78.3% of the total elderly in the town. Excluded subjects were significantly older, had more diagnosed illnesses, fewer social relationships, and a higher subsequent mortality than those not excluded (p < 0.01, data not shown).

### Statistical analysis

To evaluate gender differences in the baseline characteristics, we used chi-square and t-tests. Since age and gender differences in the association between social relationships and mortality have been well-documented,^1^^-^^5^^,^^12^^,^^15^^-^^17^ we constructed four subpopulations based on gender and two age groups; young-old (65-74 years) and old-old (75 years and older). The effect of social relationships at baseline on all-cause mortality in the subsequent 88 months was analyzed using Cox proportional hazard models with the time to death or censoring measured in months. Hazard ratios (HRs) and corresponding 95% confidence intervals (CIs) were calculated separately for the four age- and gender-based subpopulations.

As social relationship variables are more likely to be associated with each other, prior to Cox regression analyses we employed a hierarchical log-linear model, a multi-way cross-tabulation analysis often used to detect the associations among categorical variables.^18^ Highly significant two-way interactions (p < 0.001) were observed between the availability of a casual friend and a support provider for all four subpopulations. Group membership was associated with the availability of a casual friend in some of the subpopulations, and a significant association of a job with group membership was also found. Considering these significant two-way interactions among social relationship variables, we conducted two-stage Cox regression analyses. At stage 1, each of the five social relationship variables was included as a predictor in separate models, and at stage 2, all the variables were entered simultaneously in a single model, with age, diagnosed illnesses or conditions, self-rated health, annual income, and home ownership treated as common covariates for both stages. For all statistical analyses, p<0.05 was accepted as significant.

## RESULTS

During the follow-up period of 88 months, 203 men and 195 women died, for cumulative mortality rates of 24.0% and 16.6%, and a 100 person-year mortality of 3.7 and 2.4, respectively (Table 1). Both cumulative and person-year mortality were found to be significantly higher for men (p<0.001). A comparison of baseline characteristics revealed that, on average, men were significantly younger than women (73.0 vs. 73.7 years), and had less diagnosed illnesses or conditions (62.3% vs. 66.8%). In social relationships, men were significantly less likely than women to have a casual friend (81.7% vs. 86.5%) or support provider (73.2% vs. 79.4%), and to live alone (1.6% vs. 6.6%). On the other hand, men were more likely to have a job (78.3% vs. 65.5%). No significant gender difference was observed regarding group membership. As for socio-economic factors, men were more likely than women to have a higher income and to own their own homes.

**Table 1.** Baseline characteristics by gender.

	Men (n=836)		Women (n=1158)		P-value*
Mean age in years±standard deviation	73.0	±6.7	73.7	±6.5	0.022
Range: 65-97					
Number of deaths (%)	203	(24.0)	195	(16.6)	<0.001
Mortality rate (per 100 person-year)	3.7		2.4		<0.001
	
	N	(%)	N	(%)	
	
Health status					
Diagnosed illness (Yes)	521	(62.3)	773	(66.8)	0.041
Self-rated health (Fair/poor)	152	(18.2)	238	(20.6)	0.208
Socio-economic factors					
Annual income (< ¥ 1 million)	223	(26.7)	467	(40.3)	< 0.001
Home ownership (Yes)	822	(98.3)	1116	(96.4)	< 0.001
Social relationships					
Living arrangement (Alone)	13	(1.6)	77	(6.6)	< 0.001
(Couple only)	181	(21.7)	149	(12.9)	
(Multi-generation)	642	(76.7)	932	(80.5)	
Casual friend (Yes)	683	(81.7)	1002	(86.5)	0.004
Support provider (Yes)	612	(73.2)	920	(79.4)	0.001
Group membership (Yes)	654	(78.2)	898	(77.5)	0.743
Job (Yes)	655	(78.3)	758	(65.5)	< 0.001

*: P-values were calculated using *χ^2^* test for categorical data, and t-test for quantitative data.

Among the young-old, no association between social relationships and mortality was observed in either men or women at either of the two stages (Table 2). Among old-old men, having group membership and a job was significantly related to 88-month mortality at stage 1, with an HR (95% CI) of 0.53 (0.36-0.77) and 0.54 (0.37-0.78), respectively (Table 3). At stage 2, after inclusion of other social relationships, these two variables remained independent predictors of mortality with a significant HR (95% CI) of 0.62 (0.41-0.94) and 0.60 (0.40-0.90), respectively. Among old-old women, having a job was significantly associated with lower mortality as was the case among old-old men, with an HR (95% CI) of 0.65 (0.44-0.96) at stage 1 and 0.67 (0.45-0.99) at stage 2. Furthermore, old-old women who lived alone had a lower mortality risk compared to those in multi-generation households with an HR (95% CI) of 0.34 (0.13-0.94) and 0.35 (0.13-0.97). Among old-old men, a not significant but similar trend was observed.

**Table 2.** Hazard ratios (HRs) of mortality for five social relationship measures for aged 65-74 years at baseline.

Social relationships		Stage 1*		Stage 2*	
	
		HR	95% CI	HR	95% CI
		Men n=541			
Casual friend^†^		1.04	0.53 – 2.04	0.96	0.44 – 2.11
Support provider^†^		1.26	0.72 – 2.20	1.32	0.70 – 2.47
Group membership^†^		0.87	0.49 – 1.54	0.88	0.48 – 1.61
Job^†^		0.82	0.45 – 1.50	0.82	0.44 – 1.53
Living arrangement^§^	(alone)	0.97	0.13 – 7.20	0.87	0.12 – 6.50
	(couple)	0.99	0.57 – 1.72	0.96	0.55 – 1.68

		Women n=694			
Casual friend^†^		1.01	0.39 – 2.60	1.03	0.36 – 2.89
Support provider^†^		1.04	0.52 – 2.09	1.07	0.50 – 2.29
Group membership^†^		0.63	0.34 – 1.16	0.63	0.34 – 1.18
Job^†^		1.25	0.64 – 2.42	1.19	0.60 – 2.36
Living arrangement^§^	(alone)	0.40	0.09 – 1.72	0.42	0.10 – 1.84
	(couple)	1.12	0.56 – 2.25	1.01	0.49 – 2.07

CI : confidence interval

* : Stage 1 is adjusted for age, self-rated health, diagnosed illness, annual income, and home ownership. Stage 2 is adjusted for age, self-rated health, diagnosed illness, other social relationships, annual income, and home ownership.

† : To calculate the hazard ratios, “no” (coded=0) of each variable is used as a reference.

§ : To calculate the hazard ratios, multi-generation household is used as a reference.

**Table 3.** Hazard ratios (HRs) of mortality for five social relationship measures for aged 75+ years at baseline.

Social relationships		Stage 1*		Stage 2*	
	
		HR	95% CI	HR	95% CI
		Men n=295			
Casual friend^†^		0.82	0.56 – 1.22	0.90	0.56 – 1.43
Support provider^†^		1.05	0.72 – 1.53	1.16	0.74 – 1.80
Group membership^†^		0.53	0.36 – 0.77	0.62	0.41 – 0.94
Job^t^		0.54	0.37 – 0.78	0.60	0.40 – 0.90
Living arrangement^§^	(alone)	0.48	0.12 – 1.98	0.49	0.12 – 2.03
	(couple)	0.89	0.52 – 1.55	0.85	0.49 – 1.48

		Women n=464			
Casual friend^†^		0.82	0.56 – 1.23	0.92	0.58 – 1.44
Support provider^†^		0.90	0.61 – 1.33	1.01	0.66 – 1.54
Group membership^†^		0.79	0.54 – 1.15	0.88	0.59 – 1.31
Job^†^		0.65	0.44 – 0.96	0.67	0.45 – 0.99
Living arrangement^§^	(alone)	0.34	0.13 – 0.94	0.35	0.13 – 0.97
	(couple)	0.57	0.18 – 1.84	0.61	0.19 – 1.97

CI : confidence interval

* : Stage 1 is adjusted for age, self-rated health, diagnosed illness, annual income, and home ownership. Stage 2 is adjusted for age, self-rated health, diagnosed illness, other social relationships, annual income, and home ownership.

† : To calculate the hazard ratios, “no” (coded=0) of each variable is used as a reference.

§ : To calculate the hazard ratios, multi-generation household is used as a reference.

## DISCUSSION

In the present study, a significant association between social relationships and mortality emerged only among the old-old age group. While Seeman et al. observed that a significant association between social relationships and mortality showed no distinction between the two age groups of 60-69 years old and 70 years and older,^5^ another study, in which young-old and old-old city-dwelling white women were compared, concluded that benefits in terms of mortality from such social networking as participation in local organizations, contact with friends, and years lived in the area were restricted to old-old women.^10^ In this study,^10^ Yasuda et al. suggested that, with advancing age, dependence on the social environment increases to the point where the relative importance of social relationships emerges to affect the health of the old-old. In addition, we hypothesized that selective survival may have contributed in part to observed age group differences. Considering Japan’s at-birth life expectancy of 76.1 years for men and 82.2 years for women in the baseline year of 1992,^19^ the elderly who outlived others may be physically homogeneous, and therefore the relative importance of social relationships emerged in this subpopulation.

Among social relationship variables examined, availability of a casual friend or support provider failed to show any significant association with mortality among our population. Such a finding was similar to that of the Tecumseh study that observed a non-significant association of informal social relationships such as frequency of visiting friends or relatives with mortality,^2^ whereas it contradicted the Alameda findings that showed a significant inverse association between contacts or visiting frequency of friends and mortality.^1^^,^^5^ Some researchers hypothesized that, in rural areas where human interaction is so much a part of daily life, the effect of such an informal network of people might be undetected.^2^^,^^9^^,^^15^^,^^16^ Moreover, social relationships in rural areas with traditional community associations may have different connotations compared to those in an industrialized urban area. In fact, a high proportion of our study population belonged to a local organization, i.e., nearly 80% for both men and women (see Table 1), a figure higher than Japan’s national average (73.1% for men and 68.7% for women) observed in a 3-year follow-up study of 2,200 elderly persons aged 60 and over.^13^

Among the old-old men, group membership was a significant predictor of mortality. Although such an association was not significant, a similar trend was observed among other subpopulations. Studies conducted in Western nations also showed similar results.^1^^-^^5^ Supportive findings for this relation also lie in studies in Japan that reported a significant association of group membership with mortality.^13^^,^^20^

Among the old-old, those engaged in jobs were also less likely to die during the follow-up period. Studies in Japan and Taiwan demonstrated a significant association of job engagement with lower mortality among the elderly.^14^^,^^21^ A study in New Haven, Connecticut in the United States reported a similar result.^22^ One possible reason for such an association is that, for the elderly, a job can positively affect their mental health by providing more meaningful life, thus contributing to a prolonged lifespan. Our data verified that 61.5% of the old-old men with a job replied that they worked to “maintain their health or to make life meaningful and worth living,” which is one aspect of psychological wellbeing, while 44.3% of the young-old men replied the same. Among women, a similar trend was observed, with 50.0% of the old-old and 39.9% of the young-old responding similarly.

When considering the implications of having a job among the elderly, we should mention a specific characteristic of a rural society in Japan. In agricultural areas, each family member is considered an important part of the work force. Functionally able individuals are engaged in arduous jobs, whereas older people do light housework such as cleaning house or preparing meals. In such areas, the implications of being unable to work may be greater than in urban areas. In our data, 29.2% of men and 33.4% of women reported feeling isolated despite having cohabiting family members; such a tendency was observed at higher percentages among the old-old (29.7% compared to 15.3% of the young- old) and among those with illnesses (21.8% compared to 16.6% without illnesses), suggesting the negative impact of ill health on their family relationships.

Among the old-old women, those who lived alone had a lower mortality. Although the association was not significant, a similar trend was observed among old-old men. In reviews of studies on family composition and mortality, the authors reported that support from family members was not always protective against mortality, whereas support from other sources was generally associated with reduced mortality.^9^^,^^23^ In fact, a study in the United States observed that, with increasing family size, the odds of engagement in healthy behaviors such as physical activity or non-smoking decreased.^24^ Our result was in agreement with that from another Japanese study conducted in a rural area that found a significantly lower mortality among old-old women who lived alone.^25^ Moreover, suicide rate was higher among the elderly in multi-generation households according to other Japanese studies conducted in rural areas.^26^^,^^27^ Researchers suggested that inter-generational conflicts resulting from a rapid change in family values in Japan were in part attributable to such a tendency. Although marital status was not asked in our survey, women are more likely to be widowed than men because of their longer life span. It is possible that the effect of inter-generational family relationships might emerge as a relatively important factor among elderly women.

Finally, we need to address some limitations in our study. One limitation is that we could not control for all the possible confounders. Prior to this study, we eliminated the functionally dependent elderly based on self-reported ADL to minimize the confounding effect of functional impairment. However, the inclusion of information on higher level of activities such as going out using public transportation or a performance-based measure such as walking speed in the analysis might have weakened the association observed among social relationships and mortality. Because we lacked the information in this regard in our study, we could not adjust possible confounding effect of such factors. This is a limitation that should be addressed in the future.

As for a possible effect of ill health on social relations, we controlled for health status such as the number of diagnosed illnesses and self-rated health. Studies that included biological measures such as serum cholesterol and blood pressure also found a significant association of social relationships with reduced mortality.^2^^-^^5^^,^^17^ Moreover, we conducted a sub-analysis by eliminating the subjects who died within 1 year from the baseline to test that the association observed between social relationships and mortality was not a confounding effect of ill health. Our results remained unchanged. However, the inclusion of a more precise measure might have attenuated the association observed.

The effect of health behaviors or mental health on mortality was not addressed in our study either. This might be seen as another limitation. Although studies controlled for health behaviors such as smoking and physical activity found a significant association of social relationships with reduced mortality^1^^-^^5^^,^^13^ and studies controlled for depression have also observed a significant association of social relationships with lower mortality,^5^^,^^14^ we could not rule out the possible confounding effects of such factors. In addition, we used a rather crude measure of social relationships. Although many studies used similar variables and reported a significant association among them and mortality, our simple dichotomous measure might not be sufficient. The quality rather than the quantity of social interactions may be important, as indicated in reviews of social relation research.^9^^,^^23^ This may constitute another limitation that needs to be addressed in future studies.

Despite such limitations, age differences observed in the association between social relationships and mortality merit attention when considering the possible protective role of social relationships among vulnerable population, particularly the old-old. Having a job and group membership were both associated with lower mortality among old-old men, as were having a job and living alone among old-old women. The importance of social relationships may differ across different populations in culturally diverse settings. Examining the nature and source of social support may provide valuable information in further studies on social relationships and health.
